# Rehabilitation Following Hip Arthroscopy – A Systematic Review

**DOI:** 10.3389/fsurg.2015.00021

**Published:** 2015-05-26

**Authors:** Jeffrey S. Grzybowski, Philip Malloy, Catherine Stegemann, Charles Bush-Joseph, Joshua David Harris, Shane J. Nho

**Affiliations:** ^1^Hip Preservation Center, Department of Orthopedic Surgery, Rush University Medical Center, Chicago, IL, USA; ^2^Department of Physical Therapy, Marquette University College of Health Sciences, Milwaukee, WI, USA; ^3^Houston Methodist Orthopedics and Sports Medicine, Houston, TX, USA

**Keywords:** hip, arthroscopy, rehabilitation, physical therapy

## Abstract

**Context:**

Rehabilitation following hip arthroscopy is an integral component of the clinical outcome of the procedure. Given the increase in quantity, complexity, and diversity of procedures performed, a need exists to define the role of rehabilitation following hip arthroscopy.

**Objectives:**

(1) To determine the current rehabilitation protocols utilized following hip arthroscopy in the current literature, (2) to determine if clinical outcomes are significantly different based on different post-operative rehabilitation protocols, and (3) to propose the best-available evidence-based rehabilitation program following hip arthroscopy.

**Data sources:**

Per PRISMA guidelines and checklist, Medline, SciVerse Scopus, SportDiscus, and Cochrane Central Register of Controlled Trials were searched.

**Study selection:**

Level I–IV evidence clinical studies with minimum 2-year follow-up reporting outcomes of hip arthroscopy with post-operative rehabilitation protocols described were included.

**Data extraction:**

All study, subject, and surgery parameters were collected. All elements of rehabilitation were extracted and analyzed. Descriptive statistics were calculated. Study methodological quality was analyzed using the modified Coleman methodology score.

**Results:**

Eighteen studies were included (2,092 subjects; 52% male, mean age 35.1 ± 10.6 years, mean follow-up 3.2 ± 1.0 years). Labral tear and femoroacetabular impingement were the most common diagnoses treated and labral debridement and femoral/acetabular osteochondroplasty the most common surgical techniques performed. Rehabilitation protocol parameters (weight-bearing, motion, strengthening, and return to sport) were poorly reported. Differences in clinical outcomes were unable to be assessed given heterogeneity in study reporting. Time-, phase-, goal-, and precaution-based guidelines were extracted and reported.

**Conclusion:**

The current literature of hip arthroscopy rehabilitation lacks high-quality evidence to support a specific protocol. Heterogeneity in study, subject, and surgical demographics precluded assimilation of protocols and/or outcomes to generate evidence-based guidelines. Strengths and limitations in the literature were identified. Future studies should recognize and report the essentials of rehabilitation following hip arthroscopy.

## Introduction

Femoroacetabular impingement (FAI) is a common cause of pain that may lead to osteoarthritis of the hip (1). Cam and pincer FAI are two distinct anatomic entities that may lead to abnormal articular congruity and subsequent chondrolabral dysfunction (1). The acetabular labrum is an important structure in hip preservation based on improved surgical outcomes after repair vs. debridement during FAI surgery (femoral osteochondroplasty and acetabular rim trimming) (2). Early- and mid-term follow-up after FAI surgery has revealed significant improvements in hip-specific (3), general health-specific (4), and quality of life (4) questionnaires. Nevertheless, it is unknown whether FAI surgery and labral repair may prevent long-term degenerative changes of the hip (5). In addition to FAI and labral tears, several other intra- and extra-articular causes of hip pain may warrant arthroscopic/endoscopic treatment including synovial chondromatosis, loose bodies, snapping iliopsoas or iliotibial band, ligamentum teres tear, hip abductor tears, trochanteric bursitis, and proximal hamstring tear.

Rehabilitation following hip arthroscopy has long been recognized as an integral component of the clinical outcome of the procedure (6). The wide variety of bony and soft-tissue procedures precludes a standard post-operative rehabilitation for “hip arthroscopy.” Over the past decade, the incidence of hip arthroscopy has risen dramatically (7). Given the increase in quantity, complexity, and diversity of procedures performed, a need exists to define the role of rehabilitation following hip arthroscopy. The purposes of this systematic review are (1) to determine the current rehabilitation protocols utilized following hip arthroscopy in the current literature, (2) to determine if clinical outcomes are significantly different based on different post-operative rehabilitation protocols, and (3) to propose the best-available evidence-based rehabilitation program following hip arthroscopy. The authors hypothesize that (1) post-operative rehabilitation protocols are infrequently and poorly reported with significant heterogeneity, and (2) there is little to no evidence that supports or refutes specific post-operative rehabilitation protocols and that current protocols are based on theory and biomechanical, rather than clinical, investigations.

## Methods

A systematic review was conducted according to preferred reporting items for systematic reviews and meta-analyses (PRISMA) guidelines using a PRISMA checklist (8). Systematic review registration was performed using the PROSPERO International prospective register of systematic reviews (registration number CRD42013003760) (9). Two reviewers conducted the search separately on January 31, 2013 using the following databases: Medline, SportDiscus, CINAHL, and PEDro. A specific electronic search citation algorithm was utilized^1^. English language Level I–IV evidence [2011 update by the Oxford Centre for Evidence-Based Medicine (10)] clinical outcome studies with minimum 2-year follow-up were eligible. Medical conference abstracts were ineligible for inclusion. All references within included studies were cross-referenced for inclusion if missed by the initial search. Duplicate subject publications within separate unique studies were not reported twice. The studies with longer duration follow-up, greater number of subjects, or more explicit reporting of rehabilitation were retained for inclusion. Level V evidence reviews, letters to the editor, basic science, biomechanical studies, open hip surgery, imaging, surgical technique, and classification studies were excluded. Inclusive studies necessarily reported post-operative rehabilitation protocols. Qualitative and quantitative reporting of specific rehabilitation parameters was analyzed. Those studies that otherwise would have been eligible for inclusion and analysis (e.g., 2 years clinical follow-up after hip arthroscopy) that failed to include any post-operative rehabilitation protocol were excluded.

Subjects of interest in this systematic review were enrolled in a clinical trial with a minimum of 2 years follow-up following hip arthroscopy (intervention). Specific outcomes of interest regarding post-operative rehabilitation included weight-bearing status, motion, continuous passive motion (CPM), stationary bike, crutches, brace, anti-rotation boots, heterotopic ossification (HO) prophylaxis, and return to sport. Specific surgical outcomes of interest included intra- and extra-articular procedures including arthroscopic femoral osteochondroplasty, pincer acetabuloplasty, labral debridement or repair, loose body removal, articular cartilage surgery, capsular repair/plication or release, iliopsoas release, ligamentum teres debridement, gluteus medius/minimus debridement or repair, iliotibial release or windowing, and greater trochanteric bursectomy. Study and subject demographic parameters analyzed included year of publication, years of subject enrollment, presence of study financial conflict of interest, number of subjects and hips, gender, age, body mass index (BMI), diagnoses treated, and surgical procedures performed. Clinical outcome scores sought were the non-arthritic hip score (NAHS), international Hip Outcome Tool-12 (iHOT-12), hip outcome score (HOS), modified Harris hip score (mHHS), and hip disability and osteoarthritis outcome score (HOOS). Study methodological quality was evaluated using the modified Coleman methodology score (MCMS) (11). The authors declare that no financial conflict of interest influenced the topic of this manuscript.

Study descriptive statistics were calculated. Continuous variable data were reported as mean ± SD from the mean. Categorical variable data were reported as frequency with percentages. For all statistical analysis either measured and calculated from study data extraction or directly reported from the individual studies, *p* < 0.05 was considered statistically significant.

## Results

### Study, subject, and surgical demographics

**Figure 1:**
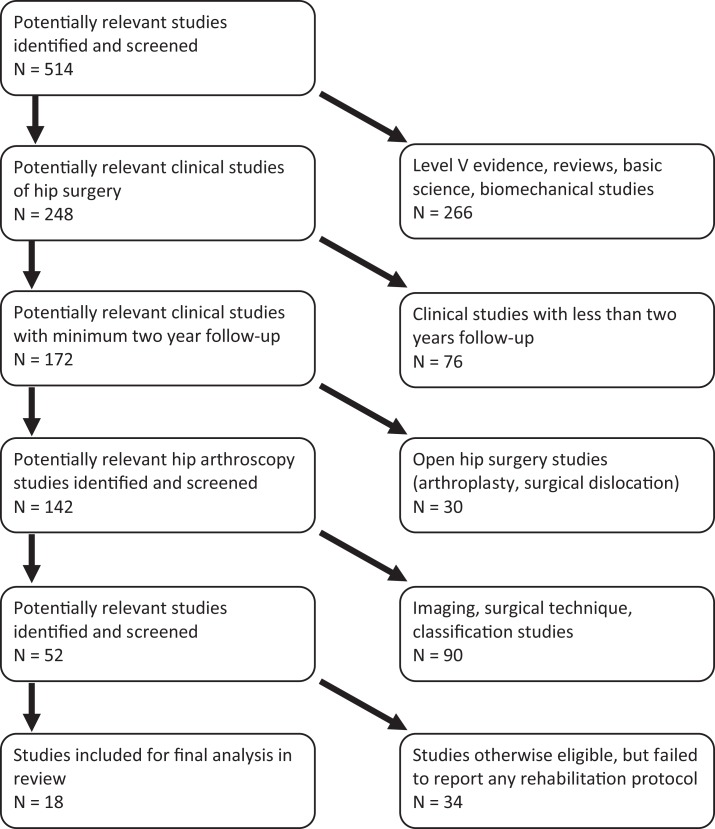
**PRISMA flowchart for selection of included and analyzed studies**.

Eighteen studies were identified for analysis (Figure 1) (3, 4, 12–27). Eligible subjects were enrolled between 1992 and 2010. Eight studies (44%) denied and five studies (28%) reported the presence of a financial conflict of interest, while five studies (28%) did not report the presence or absence of a financial conflict of interest. Fifteen studies (83%) were Level IV evidence, two (11%) were Level III, and one (6%) was Level I evidence. There were 2,092 subjects (2,099 hips) analyzed with 52% male (48% female), 48% right (52% left) hips, with mean age 35.1 ± 10.6 years (range 16.9–56.5 years) and mean BMI 24.3 ± 2.4 kg/m^2^. When present, the mean time from symptom presentation to surgery was 23.1 ± 15 months. Sixty-seven percent of surgeries (*n* = 1,408 subjects) were performed in supine position (33% lateral; *n* = 691 subjects). Mean length of follow-up was 3.2 ± 1.0 years.

Fifty-four percent (*n* = 1,127) and 80% (*n* = 1,676) of hips were diagnosed with FAI and labral tears, respectively. When reported, 67% (*n* = 634), 5.5% (*n* = 52), and 28% (*n* = 28%) were diagnosed with cam, pincer, and mixed FAI, respectively. Other primary diagnoses treated were osteoarthritis (35% of all hips; *n* = 744), ligamentum teres tear (27%; *n* = 568), chondral defects of acetabulum, femur, or both (16%; *n* = 330), loose bodies or synovial chondromatosis (5%; *n* = 98), and iliopsoas tendon pathology (3%; *n* = 62). Labral debridement was the most common surgical technique performed (66%; *n* = 1,383), followed by femoral osteochondroplasty (52%; *n* = 1,093), ligamentum teres debridement (29%; *n* = 599), acetabuloplasty rim trimming (17%; *n* = 355), labral repair (16%; *n* = 346), microfracture of femoral head and/or acetabulum (9%; *n* = 186), loose body removal (5%; *n* = 115), and iliopsoas release (3%; *n* = 62).

Mean MCMS was 33.8 ± 9.6 (poor quality). Study strengths (via MCMS) were length of follow-up, treatment description, and description of rehabilitation protocol. Study limitations were blinding, randomization, number of patients needed to treat analysis, and power analysis and alpha error calculations. MCMS question 13 (description of rehab protocol – graded 0, 2, or 4) was adequately described in 4 studies, not adequately described in 14 studies, and not described in 0 studies.

### Current rehabilitation protocols

Rehabilitation protocols were variably and poorly reported (Table 1). Allowance of immediate weight bearing as tolerated following surgery was reported in seven studies when treatment was labral debridement, synovial chondromatosis loose body removal, osteoarthritis debridement, septic arthritis debridement, and trochanteric bursectomy. When labral repair, femoral osteochondroplasty, and pincer acetabuloplasty were performed, a partial weight-bearing protocol was initiated. Three studies described partial weight bearing as “foot-flat,” while two described it as “toe-touch” or “touchdown.” Performance of microfracture warranted partial weight bearing for 4–8 weeks in four studies. Use of CPM was reported in only three studies, with between 4 and 12 h/day use for between 4 and 8 weeks. Brace/orthosis use was reported in only four studies: one study denied the use of a brace, two reported only the duration of time used (10 days, 6 weeks), and the other one did report the duration (10–21 days) and motion restrictions (prevent hip extension and external rotation) and situation (while ambulating). Anti-rotational boot use was reported in only four studies: one study denied their use, and the other three only reported the duration of time used (10 days, 2 and 3 weeks). Only five studies reported the permission and progression to return-to-sport protocols (Table 2). Initiation of low-impact sports began at 6 weeks at the earliest and high-impact sports between 3 and 6 months.

**Table 1:** **Rehabilitation protocols used in all analyzed studies**.

Study	Weight-bearing status	WBAT permitted	CPM use	Brace use	Anti-rotational boots
McDonald et al. (12)	Flat-foot WB (max 20 lbs) × 8 weeks (Mfx)	8 weeks (Mfx)	6–8 h/day × 8 weeks (Mfx)	Prevent hip extension and external rotation; 10–21 days; while ambulating	2 weeks
	Flat-foot WB (max 20 lbs) × 2 weeks (no Mfx)	2 weeks (no Mfx)	6–8 h/day × 3 weeks (no Mfx)	
Krych et al. (3)	Flat-foot PWB	2 weeks	–	–	–
McCormick et al. (13)	Flat-foot WB	Immediately post-operatively	–	–	–
Kalore and Jiranek (14)	50% WB × 1 week	1 week	–	–	–
Philippon et al. (15)	PWB × 2–3 weeks	2–3 weeks	–	–	3 weeks
Malviya et al. (4)	PWB × 4 weeks	4 weeks	–	–	–
Stafford et al. (16)	TTWB × 4 weeks	4 weeks	–	–	–
Byrd and Jones (17)	WBAT (unless Mfx, then protected × 2 months)	Immediately post-operatively (no Mfx)	–	–	–
Marchie et al. (18)	WBAT	Immediately post-operatively	–	No	No
Nho et al. (19)	20 lbs foot-flat WB × 2–3 weeks	3 weeks	4 h/day	Yes × 6 weeks	–
Haviv and O’Donnell (20)	WBAT	Immediately post-operatively	–	–	–
Horisberger et al. (21)	WBAT (unless Mfx: 15–20 kg WB for 4–6 weeks)	Immediately post-operatively (no Mfx)	–	–	–
Streich et al. (22)	Toe-touch WB 10 kg × weeks	2 weeks	–	–	–
Philippon et al. (23)	20 lbs WB (for 6–8 weeks if Mfx)	Nr	8–12 h/day × 4 weeks	10 days	10 days
Kim et al. (24)	WBAT	Immediately post-operatively	–	–	–
Fox (25)	WBAT	Immediately post-operatively	–	–	–
O’Leary et al. (26)	WBAT	Immediately post-operatively	–	–	–
Farjo et al. (27)	WBAT	Immediately post-operatively	–	–	–

**Table 2:** **Description of permission to RTS in all studies analyzed**.

Study	Permit RTS
McDonald et al. (12)	Impact sports at 3–6 months
Krych et al. (3)	–
McCormick et al. (13)	Impact loading exercises and deep squatting allowed at 4 months
Kalore and Jiranek (14)	–
Philippon et al. (15)	–
Malviya et al. (4)	–
Stafford et al. (16)	Resume pre-operative activity levels at 3 months
Byrd and Jones (17)	Impact loading allowed at 3 months
Marchie et al. (18)	–
Nho et al. (19)	–
Haviv and O’Donnell (20)	–
Horisberger et al. (21)	Low-impact RTS at 6 weeks; high-impact sports at 3 months
Streich et al. (22)	–
Philippon et al. (23)	–
Kim et al. (24)	–
Fox (25)	–
O’Leary et al. (26)	–
Farjo et al. (27)	–

Four studies (Table 3) recommended specific phase-based rehabilitation protocols following hip arthroscopy (28–31). All four studies described four phases that generally reported formal timeline-based (Table 3) and criteria-based (Table 4) protocols with precautions (Table 5) advised during each phase. Phase I was a period of protection, between 0 and 6 weeks following surgery, with limited weight bearing, restoration of early motion, limited core abdominopelvic, and hip isometric strengthening, with avoidance of excessive hip extension (beyond neutral), external rotation, deep flexion, and iliopsoas tendonitis. Phase II was a period of advancement to pain-free normal weight bearing and gait and motion, between 4 and 12 weeks post-operatively. Recommendations were for continued strengthening of core and hip muscles, while still avoiding hip flexor tendonitis. Phase III ranged between 8 and 20 weeks after surgery, with focus on endurance, in addition to strength, and progression to sport-specific training. Advancement to Phase IV generally required pain-free full motion, strength, without any subjective or objective deficits during training. Phase IV began at a minimum of 12 weeks following surgery, with progression to safe and unrestricted return to normal activities and sports as well as avoidance of any regression to pain, stiffness, or weakness. All four studies also described a permission to return to running and unrestricted sports protocols (Table 6). One study reported an explicit requirement of passage of a return-to-sport test to permit running and a different study reported an explicit requirement of passage of a test to permit unrestricted return to sports.

**Table 3:** **Phase-based description of rehabilitation protocols**.

	Phase I	Phase II	Phase III	Phase IV
Edelstein et al. (29)	0–6 weeks post-op	4–12 weeks post-op	8–20 weeks post-op	12–28 weeks post-op
	20% foot-flat WB × 2 weeks	Re-education of psoas, using eccentric exercises	Re-build strength, endurance	Improvements in explosive power
	If microfracture or gluteus medius repair, foot-flat WB 6 weeks	Re-education of transversus abdominis firing	Core control during all activities	High, low velocity strength
	No ROM restrictions unless capsular repair or iliopsoas release	Gluteal and pelvic/hip strengthening	Increase volume, intensity of aerobic activities	Sport-specific speed
	CPM × 3 weeks, brace × 10 days		Proprioception on varying surfaces, with perturbations	Repetition work
	Manual skills, soft-tissue mobilization		Plyometrics (able to squat 150% BW)	Incorporation of rest time

Wahoff and Ryan (30)	Foot-flat WB × 3 weeks (no Mfx)	Wean off crutches (depending on WB status – ±Mfx)	Continue circumduction, prone lying, soft-tissue mobilization	Return to sports
	Foot-flat WB × 6–8 weeks (Mfx)	Continue circumduction, prone lying	Gluteal activation and core and pelvis stabilization	Sport-specific training
	Brace limiting external rotation, extension × 3 weeks	Continue deep soft-tissue massage and mobilization	Double-leg strengthening advancement to single-leg strengthening	Power, plyometric, performance training
	CPM 30–70° in 10° abduction, 4-6 h/day × 2 weeks (Mfx 6–8 weeks)	Gluteal firing, core and pelvis control	Sport progressions to functional activities	
	Stationary bike 20 minutes 1–2×/day × 6 weeks	Progress cardiovascular and upper extremity fitness	Restored cardiovascular fitness	
	Circumduction 2×/day × 2 weeks; 1×/day × 10 weeks	Pilates recommended vs. yoga	Advanced power, plyometrics, performance, conditioning	
	Prone lying × 2 h/day	Reassure mental and physical rehabilitation		
	Isometrics quads, gluteus maximus, transverse abdominis	Add resistance to cycling at week 6		
	Deep soft-tissue massage			

Voight et al. (28)	Variable WB status – if biological healing required, foot-flat WB 8–10 weeks; otherwise WBAT within 1 week	Begins at week 4	Proprioceptive re-training	Return to sports
	Restore passive ROM, especially internal rotation and flexion – prevent adhesions	Pain-free full ROM	Dynamic stabilization exercises, encouraging co-contractions	Individualized based on hip pathology and surgery performed
	Stretching only to tolerance, not beyond	Continue strengthening and stabilization	Begin advanced strengthening in pool before land	
	Stationary bike without resistance	Add WB and resistance exercises	Progress exercises	
	Isometrics of gluts, quads, adductor, abductor, hamstrings, abdominals	Address muscle imbalances: tight hip flexors and erector spinae, weak gluteals and abs (forward pelvic tilt and lumbar lordosis increase)	Slow to fast Simple to complex Stable to unstable Low to high force	
	Aquatic program	Core stabilization and strengthening		

Garrison et al. (31)	Weeks 0–4	Weeks 5–7	Weeks 8–12	Weeks 12+
	50% WB for 7–10 days (unless labral repair – toe-touch WB × 3–6 weeks)	Emphasis shifts from motion to strength	Integrated functional strengthening	Safe, effective return to sports
	Flexion limited to 90° for 2 weeks (no limit extension, rotation, or abduction) for labral debridement	Continue manual therapy	Manual therapy as needed	Careful, frequent re-assessment to prevent loss of mobility as strengthening continues to advance
	Flexion limited to 90° for 2 weeks, extension to 10° for 2 weeks, rotation gentle for 2 weeks, abduction 25°2 weeks	Aquatic therapy	If full ROM not achieved by week 10, terminal stretches should be initiated	
	Prone lying 1–2 h/day	Kneeling hip flexor stretch once tolerated	Multi-planar muscle strengthening	
	Stationary bike without resistance	Passive ROM should become more aggressive, especially rotation	Core strengthening	
	Isometrics abductors, adductors, extensors, transverse abdominals	Hip and core and pelvis strengthening	Plyometrics in water	
		Add resistance to bike	Running at end of phase	
		Build cardiovascular endurance	Agility drills	

**Table 4:** **Criteria-based progression from phase to phase in post-operative rehabilitation**.

	Phase I–II	Phase II–III	Phase III–IV	Phase IV to unrestricted sports
Edelstein et al. (29)	Normalized gait without assistance	Normal ADL’s without pain	Recreationally asymptomatic	Pain-free competitive state
	No Trendelenberg	Full ROM	Maintenance of core control	Micromanagement of return to sport to consistently and painlessly perform motion responsible for initial injury
	80% full ROM	Core stability Sahrmann 2 × 30 s	“10 rep triple”	
	Core stabilization	5/5 manual muscle strength		
		Good control in single-leg squat		

Wahoff and Ryan (30)	Minimal pain with all Phase I	Pain-free normal gait	Passing of a sports test, allowing return to practice without limitations	Physician clearance
	Minimal “pinching” before 100° flexion	Full ROM Core, pelvic stability Balance, proprioception	Perform all Phase III exercises pain free and with correct form	Full return to practice without restrictions
	Tolerated full WB			

Voight et al. (28)	Close to full ROM	Pelvic tilt test, pelvic rotation test, torso rotation test, bridge with leg extension test	Proprioceptive and neuromuscular control	Depends on hip pathology treated and specific demands of sport played
	Normalized gait without crutches	
	Minimal to no pain	

Garrison et al. (31)	ROM ≥ 75% contralateral side	Normal gait without Trendelenberg sign	Symmetric motion	Completion of return-to-play test using sportcord test
	Ability to do side-lying straight-leg raise	Symmetric passive ROM	Symmetric flexibility of psoas, piriformis	Dynamic functional activities with resistance from sportcord: single-leg squat × 3 min, lateral bounding × 80 s, forward/backward jogging × 2 min
		No pain	No Trendelenberg with higher level functional strengthening	

**Table 5:** **Precautions recommended at each phase in post-operative rehabilitation**.

	Phase I	Phase II	Phase III	Phase IV
Edelstein et al. (29)	Not lifting leg on its own	Avoid hip flexor tendonitis (iliopsoas, TFL, sartorius, rectus femoris)	Avoid sacrificing quality for quantity during strengthening	Avoid breakdown to acute inflammatory response
	Not crossing legs	
	Not pushing ROM to point of pain	

Wahoff and Ryan (30)	No hip extension past neutral × 3 weeks	Avoid treadmill (shear stress)	Avoid treadmill	None
	No external rotation × 3 weeks	Avoid hip flexor and adductor inflammation	Avoid hip flexor and adductor inflammation	
	No flexion beyond 120°	Avoid ballistic stretching	Avoid ballistic stretching and high-velocity activities	
	No abduction beyond 45°			

Voight et al. (28)	No recumbent bike	Avoid arthrokinetic inhibition	Depends on tolerance to advancement of activities	Avoid compressive forces generated by sports, depending on hip pathology and surgical treatment
	No aerodynamic bike riding position	Avoid synergistic dominance	
		Avoid reciprocal inhibition	

Garrison et al. (31)	Avoid tight hip flexors and erector spinae	Avoid pain	Avoid any loss of motion	Avoid loss of flexibility as strength continues to increase
	Avoid inhibited gluts and abs		Avoid loss of core strength	
	Avoid hip flexion straight-leg raises to avoid hip flexor tendonitis			

**Table 6:** **Criteria-based permission to return to running and return to sports described in each study**.

	Permission to run	Unrestricted sports
Edelstein et al. (29)	“10-rep triple”: 10 front step-downs and 10 single-leg squats without kinetic collapse, 10 side-lying leg raises against resistance with at least 4/5 manual muscle strength	Consistent and painless repetitions of the movement responsible for the mechanism of injury
Wahoff and Ryan (30)	Pain-free, progressive, predictable	Physician clearance after return to unrestricted practice
	Initiate pool running several weeks prior to land in runners	
Voight et al. (28)	Not reported	Depends on hip pathology and surgical treatment performed
Garrison et al. (31)	Pool running at 2–3 weeks	Completion of return-to-play test using sportcord test – Dynamic functional activities with resistance from sportcord: single-leg squat × 3 min, lateral bounding × 80 s, forward/backward jogging × 2 min
	Once good eccentric control, muscular endurance, ability to generate power	

### Clinical outcomes

Clinical outcomes were variably and poorly reported (Table 7). Significant improvements were demonstrated for multiple diagnoses treated with various surgical techniques utilizing NAHS, HOS, HOOS, and mHHS. However, given the heterogeneity between subjects and surgeries performed, no comparison could be made between any group of subjects based on the rehabilitation protocol following surgery.

**Table 7:** **Salient outcomes in all studies analyzed**.

Study	Level of evidence	Subject population	Study design	Intervention	Primary outcome
McDonald et al. (12)	3	Elite athletes	Case-control	Microfracture (case) vs. no microfracture (control)	• Return to sport: 77% in microfracture vs. 84% in non-microfracture (*p* > 0.05)
Krych et al. (3)	1	Females	RCT	Labral repair vs. debridement	• Better HOS (ADL, sport) in repair group (*p* < 0.05 for both)
					• Better subjective outcome in repair group (*p* < 0.05)
McCormick et al. (13)	3	Patients with labral tears	Case-control	Labral repair vs. debridement	• Presence of OA at arthroscopy predictive of worse outcomes
					• Age >40 years predictive of worse outcomes
Kalore and Jiranek (14)	4	Patients with labral tears	Case series	Labral repair vs. debridement	• Higher (*p* < 0.05) re-operation rate in
					○ Borderline vs. adequate acetabular coverage
					○ Labral debridement vs. repair
Philippon et al. (15)	4	FAI, 11–16 years of age	Case series	FAI and labral treatment	• Significant (*p* < 0.05) improvement in mHHS (57–91 at 3 years)
					• 8/60 (13%; all girls) needed repeat arthroscopy (adhesions)
Malviya et al. (4)	4	FAI, 14–75 years of age	Case series	FAI and labral treatment	• Significant (*p* < 0.05) improvement in QoL
					• 74% of patients happy with results
Stafford et al. (16)	4	FAI, chondral defect acetabulum	Case series	Microfracture with repair of delaminated cartilage using fibrin adhesive	• Significant (*p* < 0.001) improvement in mHHS at 2 years
Byrd and Jones (17)	4	FAI	Case series	FAI and labral treatment	• Significant (*p* < 0.001) improvement in mHHS at 2 years
Marchie et al. (18)	4	Synovial chondromatosis	Case series	Loose body removal	• 48% good/excellent outcomes at 5.3 years
					• 17% underwent total hip arthroplasty at mean 4.3 years
Nho et al. (19)	4	High-level athletes, FAI	Case series	FAI and labral treatment	• Significant improvements in mHHS and HOS at 2 years
					• 79% return to sports at mean 9.4 months
Haviv and O’Donnell (20)	4	Osteoarthritis	Case series	FAI and labral treatment	• 16% of patients eventually underwent total hip arthroplasty
					• Age <55 years and mild osteoarthritis predictive of longer time to arthroplasty
Horisberger et al. (21)	4	Osteoarthritis	Case series	FAI and labral treatment	• 40% of patients eventually underwent total hip arthroplasty
					• Mean index time to arthroplasty was 1.4 years (range 0.4–2.2)
Streich et al. (22)	4	Labral tears, no FAI	Case series	Labral treatment	• Significant improvements in Larson hip score and mHHS
					• Presence of acetabular chondral defect worse prognosis
Philippon et al. (23)	4	FAI, 38–44 years of age	Case series	FAI and labral treatment	• Significant improvements in mHHS at 2 years
					• 11% of patients underwent total hip arthroplasty at mean 16 months
Kim et al. (24)	4	Septic arthritis	Case series	Arthroscopic debridement and drainage	• Excellent results obtained at 4.9 years
					• No complications, no re-operations
Fox (25)	4	Trochanteric bursitis	Case series	Trochanteric bursectomy	• 85% excellent/good results at 5 years; 96% satisfaction
					• Only 2 recurrences of pain
O’Leary et al. (26)	4	Various	Case series	Various arthroscopic techniques	• 60% significant improvements at 2.5 years
					• OA and AVN had significantly worse outcomes (vs. labral tears)
					• 21% underwent total hip arthroplasty at mean 8.4 months
Farjo et al. (27)	4	Labral tear	Case series	Labral debridement	• 46% good, 54% poor results
					• 29% underwent total hip arthroplasty at mean 23 months

## Discussion

The purposes of this systematic review were to determine the current rehabilitation protocols utilized following hip arthroscopy in the current literature, if clinical outcomes are significantly different based on different post-operative rehabilitation protocols, and to propose the best-available evidence-based rehabilitation program following hip arthroscopy. The authors hypothesized that post-operative rehabilitation protocols are infrequently and poorly reported with significant heterogeneity. The authors also hypothesized that there is little to no evidence that supports or refutes specific post-operative rehabilitation protocols and that current protocols are based on theory and biomechanical, rather than clinical, investigations. The study hypotheses were confirmed, thus strengthening the previous assertion by Cheatham et al. that there is a paucity of evidence surrounding post-operative rehabilitation protocols following hip arthroscopy (32).

Rehabilitation following hip arthroscopy is an integral part of a successful outcome in treatment of various intra- and extra-articular hip pathologies. The current medical climate mandates assimilation of evidence-based medicine and patient-reported outcomes into everyday clinical practice. This includes assessment of basic science and clinical outcomes literature and incorporation of this evidence into discussions with patients. This mandates that the rehabilitation literature following hip arthroscopy significantly improve. The authors selected clinical follow-up studies with minimum 2-year follow-up to accurately identify current rehabilitation protocols. Although 18 studies were identified for inclusion and analyzed, nearly twice as many studies (*n* = 34) would have also been included (Figure 1), but those studies did not report a single word about rehabilitation in the entirety of the study. Even within the 18 studies included for final analysis, evaluation of the quality of their reporting was poor (via MCMS) and significant heterogeneity was demonstrated. Little recognition of the importance of rehabilitation was exhibited in the current literature. This does not necessarily mean that the quality of rehabilitation or the conduct of the trial is poor, only that the quality of reporting is poor.

Given the inability to extract evidence-based guidelines from clinical outcome studies of hip arthroscopy rehabilitation in this systematic review, the authors utilized narrative review articles (Tables 3–6) to summarize and report the best-available evidence on the topic.

### Principles of rehabilitation

Rehabilitation following hip arthroscopy should be individualized and evaluation based rather than time based. Circumduction is key in enhancing early motion and preventing intra- and extra-articular adhesions. Weight bearing and motion progression is based upon the specific surgical techniques performed. Thus, a “cookbook” rehabilitation program after arthroscopic surgery of the hip is not recommended. Nevertheless, when protection or biological healing is required (labral repair, capsular repair or plication, femoral osteochondroplasty), rehabilitation should progress more slowly vs. procedures in which no protection or healing is needed (labral debridement, loose body removal, ligamentum teres debridement, synovectomy). Avoidance of hip flexor tendonitis is recommended throughout rehabilitation [not only primary hip flexors (iliopsoas) but also secondary flexors (rectus femoris, sartorius, tensor fascia lata)]. Given that the iliopsoas is largely inhibited early after surgery, the activation and over-activation of secondary flexors may occur, thus relegating them to potential inflammatory overuse.

Patients undergoing hip arthroscopy are young (mean age 35 years in this review) and active. As such, rehabilitation protocol efficacy should be assessed using patient-reported outcome instruments that are appropriate for use in this patient population. HOS, the International Hip Outcome Tool (iHOT-33/iHOT-12), and the Copenhagen hip and groin outcome score (HAGOS) have been recommended to guide therapy progression (33). Wahoff et al. described a comprehensive, criteria-driven algorithm for safe integration and return to sport rehabilitation following hip arthroscopy. Emphasis is placed on various criteria to advance through the six phases including healing restraints, patient-reported outcomes, range of motion, and other sport-specific tasks. As a part of the minimum criteria for advancement, the HOS was chosen as it contains both ADL and sports subscales. These separate scales make it appropriate for use in both early rehabilitation and late as it is responsive during higher levels of physical ability (34).

Return to sport is a very relevant component of the surgical outcome. Too early return may lead to recurrence of pain. Progression through phases of rehabilitation necessitates meeting specific goals and milestones as described above. Passing these thresholds improves the likelihood of safe return to sport. Return-to-sport tests are gaining acceptance in return to play following ACL reconstruction (35, 36). The same standards should be applied to patients undergoing hip arthroscopy, as the subject demographics, rehabilitation timelines, and sport goals are similar.

### Limitations

The limitations of any systematic review are dependent upon the included studies, which it analyzes. Selection bias in this review was minimized by the inclusive nature of study selection. However, bias is also recognized by exclusion of studies with <2 years follow-up. Performance bias was also minimized by the inclusive nature of study selection, allowing all subject diagnoses and surgical treatments available to be included. It is recognized, however, that no study reported subject compliance with rehabilitation, including weight-bearing status, motion restrictions, CPM use, brace or boot use, or return to sports. Heterogeneity in definitions of rehabilitation phases, protocols, goals, precautions, and return to sport variables introduces detection bias. Study design bias is present in the retrospective nature of 17 out of 18 (94%) included studies. Publication bias is present in that the authors excluded medical conference abstracts, non-English language studies, and non-published English language studies.

## Conclusion

The current literature of hip arthroscopy rehabilitation lacks high-quality evidence to support a specific protocol. Heterogeneity in study, subject, and surgical demographics precluded assimilation of protocols and/or outcomes to generate evidence-based guidelines. Strengths and limitations in the literature were identified. Future studies should recognize and report the essentials of rehabilitation following hip arthroscopy.

## Conflict of Interest Statement

Shane J. Nho is a paid consultant for Stryker, Pivot Medical, and Ossur; owns stock in Pivot Medical; and receives research support from Arthrex, Linvatec, Smith and Nephew, DJ Orthopaedics, Miomed, Athletico, Stryker, Pivot Medicine, and Allosource. Joshua David Harris is on editorial board for Arthroscopy: The Journal of Arthroscopic and Related Surgery; is a paid consultant for NIA Magellan; and receives royalties from SLACK, Inc. Charles Bush-Joseph is an unpaid consultant for The Foundry and is on the Medical Publications editorial/governing board for the American Journal of Sports Medicine. All other authors have no significant financial conflict of interest. However, the authors confirm that no financial conflict of interest influenced the topic of this manuscript.

## Footnotes

^1^(((((((((((((((((((arthroscopy[Title/Abstract]) AND hip[Title/Abstract])) NOT shoulder[Title/Abstract]) NOT elbow[Title/Abstract]) NOT wrist[Title/Abstract]) NOT knee[Title/Abstract]) NOT ankle[Title/Abstract])) NOT lumbar[Title/Abstract]) NOT lumbosacral[Title/Abstract]) NOT sacrum[Title/Abstract]) NOT sacroiliac[Title/Abstract]) NOT sacral[Title/Abstract])) NOT cadaver[Title/Abstract]) NOT cadaveric[Title/Abstract]) NOT biomechanical[Title/Abstract])) NOT revision[Title] AND (English[lang]).
